# Burkitt Lymphoma of the Duodenum: An Uncommon Phenomenon

**DOI:** 10.1155/2019/7313706

**Published:** 2019-03-07

**Authors:** Fahad Malik, Manuel Gonzalez, Wahib Zafar

**Affiliations:** Internal Medicine, Richmond University Medical Center, Staten Island, NY 10310, USA

## Abstract

Burkitt lymphoma is an aggressively growing tumor commonly found in African children, involving the jaw and facial bones. Most non-Hodgkin lymphoma tumors involve extra nodal sites like the nervous system and gastrointestinal tract. A rare variant of this type of lymphoma is found in immunocompromised patients specifically in the gastrointestinal tract with accompanying gastrointestinal symptoms. Burkitt lymphoma is a malignancy that has commonly presented in GI tract but rarely in the duodenum. This clinical variant can commonly involve stomach, ileum, and cecum. However, there is very limited data available regarding the duodenal growth of this tumor. Duodenal involvement of Burkitt lymphoma is extremely rare and accounts for < 1% of all lymphomas. We present a case report of an older patient with a duodenal Burkitt lymphoma diagnosed by biopsy. A high suspicion should be present while treating immunocompromised patients with chronic abdominal symptoms especially with complications such as bleeding or occult positive testing. Early endoscopy intervention with biopsy can help identity and treat these conditions with improved outcomes for the patients.

## 1. Introduction

Burkitt lymphoma is an aggressively growing tumor commonly found in African children, involving the jaw and facial bones. A rare variant of this type of lymphoma is found in HIV positive or immunocompromised patients specifically in the gastrointestinal tract with accompanying gastrointestinal symptoms. This clinical variant can commonly involve stomach, ileum, and cecum. However, there is limited data available regarding the duodenal growth of this tumor. Duodenal involvement of Burkitt lymphomas is extremely rare and accounts for < 1% of all lymphomas. We present a case of an older adult with duodenal Burkitt lymphoma diagnosed by biopsy due to the chronic abdominal pain.

## 2. Case History

A 39-year-old male, with no significant previous medical history, presented to the emergency department for evaluation of severe diarrhea. He complained of continued lightheadedness, severe abdominal tenderness, watery diarrhea with semi-formed dark stools five to seven times daily, and unintentional weight loss of about twenty pounds in the past three months due to loss of appetite. The patient described the severity of the abdominal pain as 10/10, intermittent, burning, and located in the right lower quadrant and having no correlation with food intake. He was a nonsmoker and nonalcoholic but admitted to binge drinking at times on the weekends. He also admitted to having unprotected sex occasionally with multiple female partners in the past few years. He worked as a full time employee in a restaurant. The family history revealed carcinoma in the grandmother which was not worked up.

Physical examination revealed a young looking male of average build resting comfortably in bed. Lung examination revealed no abnormalities and cardiac examination was normal. Skin examination was unremarkable. The patient had no palpable lymph nodes. Abdominal examination revealed mild epigastric tenderness on deep palpation but no hepatosplenomegaly or rebound tenderness. Rectal examination revealed normal sphincter tone with heme occult positive stool.

Abnormal laboratory findings included the following: complete blood count, hemoglobin 6.6g/dl; hct 20.7%; MCV 89.1 fl; on chemistry, Na 134 mmol/L; chloride 107 mmol/L; lactate dehydrogenase 1060 U/L; bicarbonate 25 mmol/L; anion gap 13.2;AST 59 u/l, ALT 30 u/l, uric acid 10.5 mg/dl, total protein 7.3 g/dl; albumin 1.9g/dl; vitamin B12 >2000 pg/mL; iron studies revealed microcytic anemia with iron 16mcg/dl; TIBC 213mcg/L; % saturation 7.5%; transferrin 163 mg/dL; ferritin 26 ng/mL; white blood count, platelets, potassium, magnesium, phosphorus, renal function, lactic acid, alkaline phosphatase, total bilirubin, direct bilirubin, and lipase were normal; urinalysis, urine cultures, and blood cultures x2 were negative. HIV-1 antibody was positive. This was a new diagnosis for the patient. CD4 count was 126 cells/mcl.

**Figure 1:**
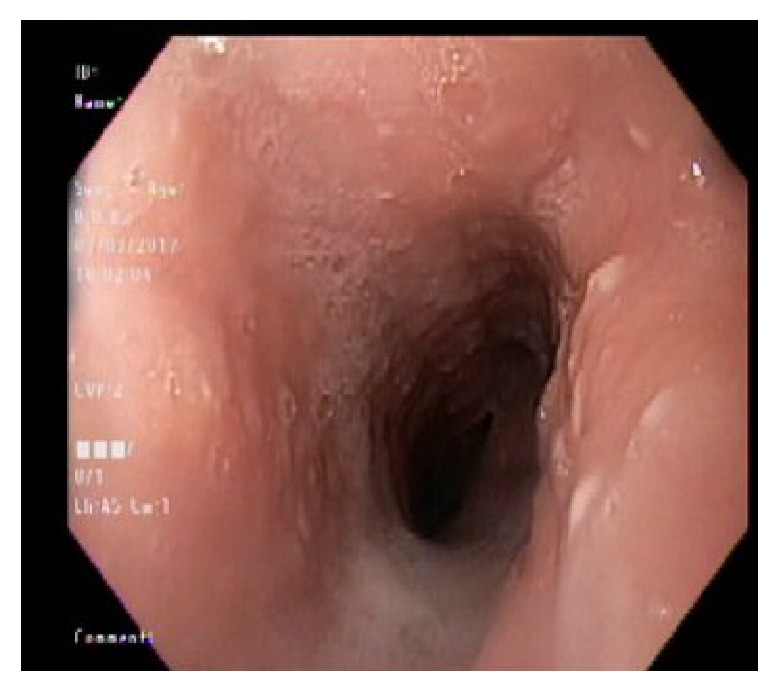
Candidiasis.

**Figure 2:**
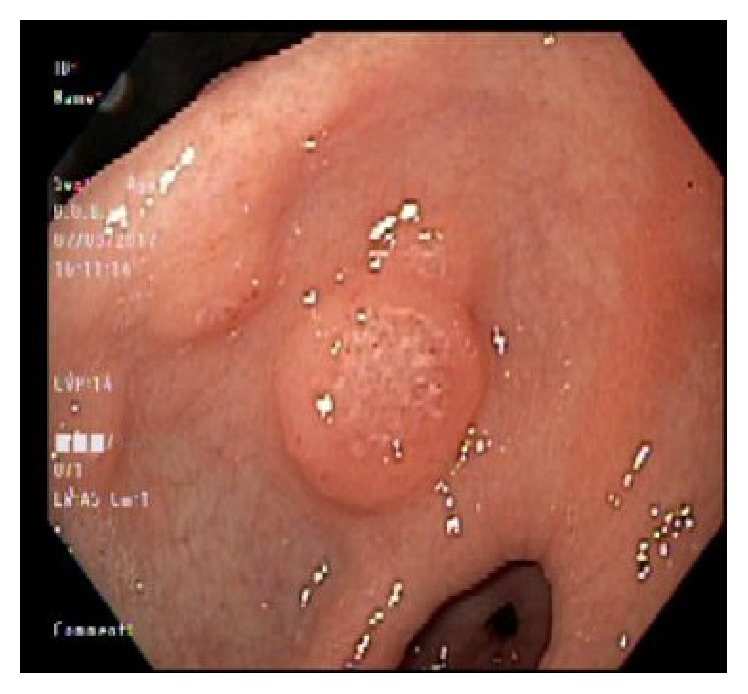
Ulcerated nodule in antrum.

**Figure 3:**
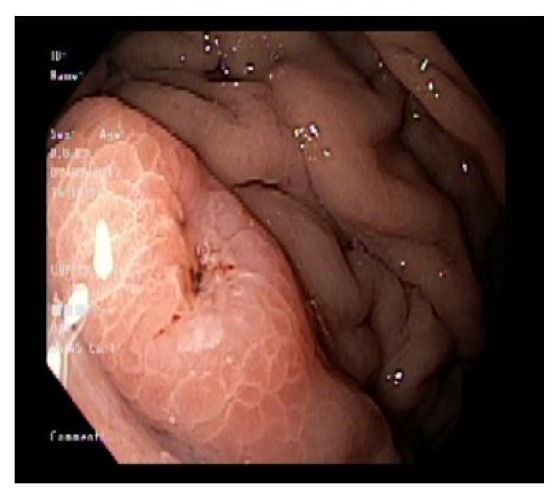
Ulcerated nodule in the gastric body.

**Figure 4:**
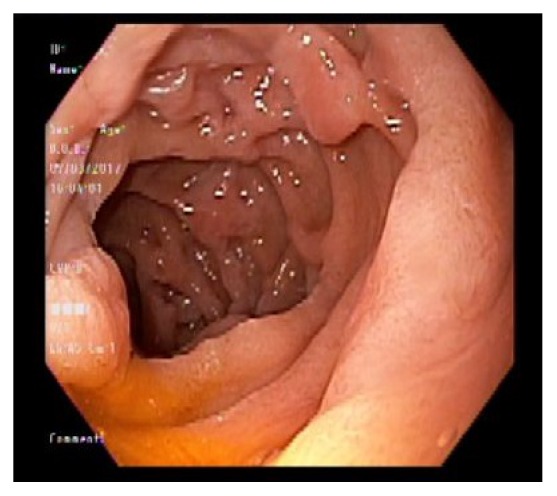
Nodules in the 2nd part of duodenum.

Chest X-ray and ultrasound of the abdomen were essentially normal. Esophagogastroduodenoscopy showed esophageal candidiasis (Figure 1) and multiple nodules in the antrum (Figure 2), along with nodules in the body of the stomach with central erosions and nonbleeding ulcerations. One large nodule found to be in the body of the stomach with central ulceration which was healing and nonbleeding at the time. This was assumed to be the culprit of iron deficiency microcytic anemia (Figure 3). The second part of duodenum was filled with multiple nodules without erosions or active bleeding (Figure 4). Pathology of the gastric mucosa showed atypical lymphoid proliferation which is typical for reactive gastropathy. The biopsy of the duodenum was consistent with Burkitt lymphoma.

The pathology report revealed that the small intestinal mucosal region was infiltrated by multiple clusters of atypical B-lymphocytes. These atypical lymphocytes cells were positive for CD20, CD79a, PAX5, CD10 weakly, BCL6, and cMYC and negative for MUM1, BCL2, CD5, CD23, CD30, cyclin D1, CD138, kappa, and lambda. CD20, PAX5, and CD79 positivity indicated a B-cell lineage origin. MYC and BCL6 positivity along with negative BCL2 markers indicated oncologic cell proliferation unlikely from benign hyperplasia. The proliferation index was calculated to be 90-100% for ki67/mib1 indicating a very high proliferation rate. This report also included mitotic figures and multiple focal macrophages. Fluorescence in situ hybridization results interpretation was also consistent with 68% of the cells having a positive signal pattern with MYC-IGH [t (8; 14)] fusion. The hallmark of Burkitt lymphoma is the presence of MYC-IGH rearrangement. This report was strongly consistent with Burkitt lymphoma.

The patient was initially treated with Fluconazole for candidiasis and pantoprazole for ulcerations. The patient underwent a complete work-up including a bone marrow biopsy, lumbar puncture, and complete body imaging for staging purposes along with further work-up for acquired immune deficiency syndrome. The patient was diagnosed with Burkitt lymphoma of stage IV-B as per Ann Arbor staging system for lymphoma grading due to lymphocytic infiltration to bone marrow. The HAART regimen (Truvada/Raltegravir) and prophylactic treatment for opportunistic organisms began once the CD4 count of 126 resulted. An Ommaya reservoir shunt and port was placed for the patient to begin chemotherapy. He was treated rophylactically with allopurinol to prevent tumor lysis syndrome. He was started on an anthracycline based chemotherapy regimen. Intrathecal methotrexate was started as part of R-hyper-CVAD (Rituximab, Cyclophosphamide with Mesna, Dexamethasone, Doxorubicin, and Vincristine) and Neupogen was given as required. The patient continued having multiple episodes of diarrhea while undergoing inpatient chemotherapy treatment which began resolving within 3 days of chemotherapy and the patient finally began to have an improved quality of life. The patient was discharged after a total of 12 days from the hospital and currently continues to follow up for outpatient chemotherapy. He was scheduled for a colonoscopy to complete gastrointestinal work-up on multiple occasions but due to neutropenia from chemotherapy it had to be postponed.

## 3. Discussion

Lymphomas are commonly grouped as Hodgkin's lymphomas or non-Hodgkin's lymphomas. Of all the adult non-Hodgkin's lymphomas, only 1-2% are Burkitt lymphoma [1, 2]. Burkitt lymphomas are further classified into endemic, sporadic, or immunocompromised related Burkitt lymphoma. Sporadic Burkitt lymphoma accounts for 1-2% of all adult lymphomas [3, 4]. The immunodeficiency related Burkitt lymphoma usually is held accountable for over 30% of lymphomas in immunocompromised patients but very few cases have ever been reported specifically in the duodenal region of the gastrointestinal tract. Burkitt lymphoma is known to be an aggressive B-cell lymphocytic malignant tumor that has commonly presented in the gastrointestinal tract mainly in stomach but rarely in the duodenum [5–7].

The hallmark of Burkitt lymphoma is the presence of MYC-IGH 8:14 rearrangements. Our patient demonstrated negative kappa and lambda chains but flow cytometry immunophenotyping can have multiple variations in Burkitt lymphoma. Therefore, the MYC-IGH 8:14 rearrangements are diagnostic even in the event of having lack of light chain expressions [8–10]. Burkitt lymphoma is well known to be an aggressively growing tumor; nonetheless, if appropriately treated with HAART and chemotherapy, it has shown to quickly respond to treatment. HAART and chemotherapy (hyper-CVAD and Methotrexate) combined can significantly improve outcomes in patients like in our patient. It is still uncertain whether this regimen would improve the overall prognosis for immunocompromised related Burkitt lymphoma [11, 12].

Across all variants, Burkitt lymphoma of the duodenum the immunocompromised subtype is extremely rare and very limited literature is currently present. Duodenal involvement of Burkitt lymphomas likely accounts for less than 1% of all cases as there are only a few cases to be studied for reference [1–3]. In conclusion, a high suspicion of lymphomas should be present while treating immunocompromised patients with chronic abdominal symptoms especially with complications such as bleeding or occult positive testing. Early endoscopy intervention with biopsy can help identify these conditions and improve outcomes for the patients [2, 5, 7]. Burkitt lymphoma is a malignancy that has commonly presented in gastrointestinal tract but rarely in the duodenum. As survival rates of AIDs patients improve due to more effective antiviral treatments, more cases of duodenum lymphomas will be described in literature, and we can better assess the implications of lymphomas according to anatomical location.
